# Stepwise Incremental Dose Schedule of Sirolimus Is Successfully Tolerated by a Patient With Lymphangioleiomyomatosis Who Was Initially Allergic to mTOR Inhibitors

**DOI:** 10.7759/cureus.58805

**Published:** 2024-04-23

**Authors:** Kuniaki Seyama, Etsuko Komiyama, Hitoshi Tsuchihashi, Makiko K Okura, Yasuhito Sekimoto, Yoichiro Mitsuishi

**Affiliations:** 1 Respiratory Medicine, Juntendo University Faculty of Medicine and Graduate School of Medicine, Tokyo, JPN; 2 Dermatology, Juntendo University Faculty of Medicine and Graduate School of Medicine, Tokyo, JPN

**Keywords:** tumor suppressor gene syndrome, sirolimus, mtor inhibitors, molecular targeting therapy, desensitization, allergic cutaneous reactions

## Abstract

Lymphangioleiomyomatosis (LAM) is a rare disease involving the proliferation of LAM cells in the lungs and the axial lymphatic system and mechanistic target of rapamycin (mTOR) inhibitors are the only effective medicines for treating it. Patients suffering from LAM, who are allergic to mTOR inhibitors can be treated by desensitizing them to the medicine.

A 39-year-old woman presented with dyspnea caused by chylous pleural effusion, ascites, and retroperitoneal lymphangioleiomyomas. She was diagnosed with LAM based on the presence of LAM cell clusters (LCCs) in chylous pleural effusion and elevated serum vascular endothelial growth factor D (VEGF-D) concentration. She was allergic to cedars and yellowtails. Although she was started on sirolimus for treating LAM, the drug had to be discontinued on day 45 because of the appearance of a skin rash on her trunk. A year later, another oral mTOR inhibitor, everolimus, was initiated but had to be discontinued because of the appearance of cutaneous reactions. Since mTOR inhibitors are the only effective molecular-target medicines for LAM, desensitization to sirolimus was attempted by initiating exposure to sirolimus at a low dose followed by stepwise dose escalation. Eventually, the patient tolerated a dose of 0.5 mg/day of sirolimus, which resulted in a trough concentration of approximately 2 ng/ml in blood, without adverse cutaneous reactions; furthermore, clinically relevant effects were observed as her LAM condition reduced and stabilized.

This case study illustrates that mTOR inhibitor therapy for LAM should not be abandoned because of allergic cutaneous reactions. Physicians must find a dose that balances adverse events and therapeutic effects to ensure continued treatment for patients with LAM. Furthermore, the possible mechanisms for mTOR inhibitor-induced cutaneous reactions have been discussed.

## Introduction

Lymphangioleiomyomatosis (LAM) is a rare disease involving the proliferation of LAM cells that resemble smooth muscle cells, in the lungs and axial lymphatic system. The LAM cells nodularly proliferate in the lungs and form numerous cysts. In advanced cases, LAM can result in respiratory failure. Loss-of-function-type mutations in the tumor suppressor genes TSC1 or TSC2 result in the formation of the neoplastic LAM cells, thereby dysregulating the mechanistic target of rapamycin (mTOR) [1]. The Multicenter International LAM Efficacy of Sirolimus (MILES) trial, a randomized and double-blind study investigating the efficacy and safety of sirolimus for LAM, demonstrated that sirolimus, an mTOR inhibitor, suppressed the decline of lung function [2].

Currently, the mTOR inhibitors sirolimus and everolimus, are the evidence-based and first-line drugs for treating LAM. However, the mTOR inhibitors exert cytostatic effects against LAM cells, because of which their efficacy is considered to be disease-modifying and not curing, and LAM patients are required to take mTOR inhibitors for extended periods of time. On the other hand, as the disease almost exclusively affects women, anti-estrogen therapy has been used as an empiric therapy in women; LAM cells express estrogen and progesterone receptors, and the disease can progress during pregnancy. The efficacy of anti-estrogen therapy for LAM has not been proven in clinical trials [3]. However, a longitudinal analysis of the National Heart, Lung, and Blood Institute (NHLBI) LAM Registry data recently demonstrated that menopausal status significantly affected the rate of forced expiratory volume (FEV1) decline and progression of LAM [4], indicating that estrogen-depleting treatment might play a role in the treatment of LAM.

An mTOR is a ubiquitously expressed protein that plays a significant role in maintaining cellular homeostasis (growth, proliferation, and metabolism, etc.) in response to extracellular stimuli and internal nutritional conditions [5]. Therefore, adverse events such as stomatitis, acne-like skin rash, upper respiratory tract inflammation, and dyslipidemia have been reported [2,6]. Furthermore, hypersensitivity reactions to mTOR inhibitors and successful rapid oral desensitization to mTOR inhibitors have recently been reported [7,8]

We report the case of a patient diagnosed with LAM, who developed adverse cutaneous hypersensitivity reactions to sirolimus and everolimus, necessitating the discontinuation of mTOR inhibitors. A chemical oophorectomy with a gonadotropin-releasing hormone analogue (GnRH) was performed as the second-best treatment. Although a certain level of symptomatic improvement was observed, the magnitude of the effect was not sufficient to control LAM. Because of the lack of other medications that have been backed by clinical studies for stabilizing LAM, the decision to desensitize the patient to sirolimus was taken. Instead of an oral rapid desensitization protocol, the method involving a slow increase in the exposure to sirolimus by initiating a low dose in the setting of an outpatient clinic to induce desensitization was chosen.

## Case presentation

**Figure 1:**
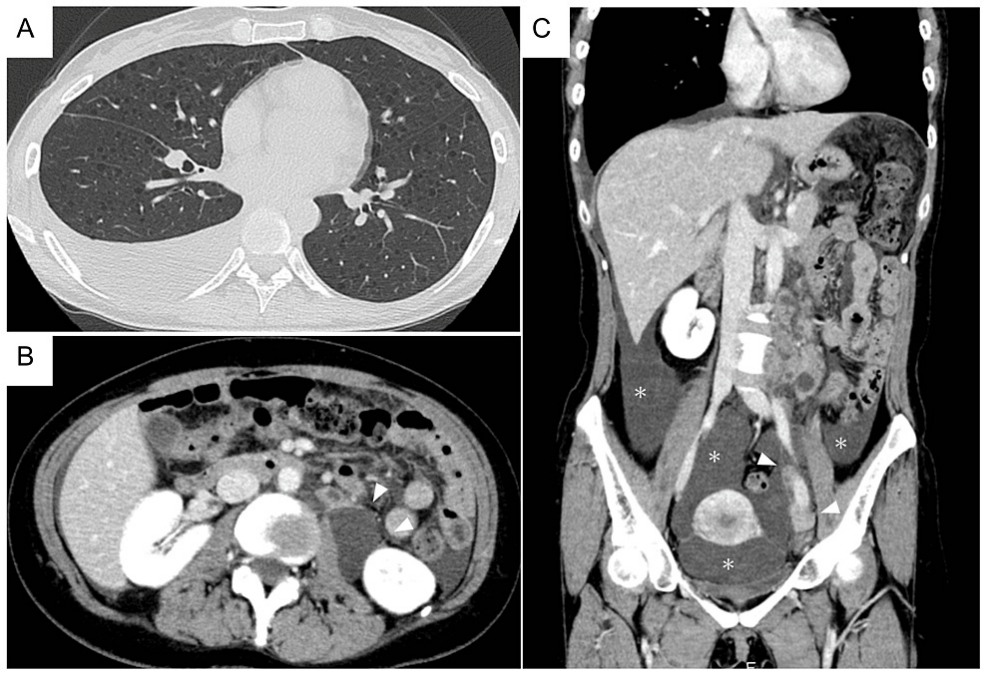
CT images of the chest, abdomen and pelvic cavity at the initial presentation A: Axial view of the lung; B: Axial view of kidneys; C: Coronal reconstruction image from the abdomen to the pelvic cavity Both B and C are contrast-enhanced images. Multiple thin-walled, small round cysts were observed to be diffusely scattered in both lungs with a moderate level of right pleural effusion. Lymphangioleiomyomas with a partly cystic appearance were observed in the left paraaortic near to left external iliac artery area (indicated by arrowheads in B and C). A moderate amount of ascites was also noted (* in C).

A 39-year-old woman experienced exertional dyspnea and abdominal distention after undergoing fertility treatment for approximately three months. The presence of right chylous pleural effusion and ascites, multiple thin-walled cysts in both lung fields, and tumorous shadows that suggested retroperitoneal lymphangioleiomyomas from the upper abdomen to pelvic cavity indicated the presence of LAM (Figure 1).

**Figure 2:**
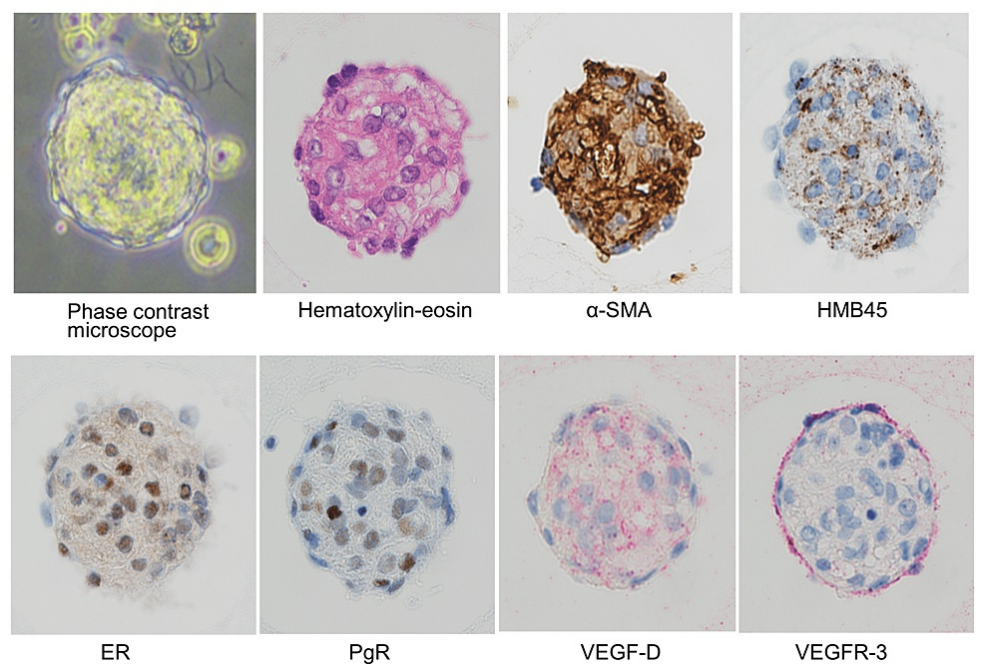
LCCs were identified in the right chylous pleural effusion The LCCs were identified in chylous pleural effusion under a phase contrast microscope. They were collected, embedded in iPGell (Nichirei Co. Ltd., Tokyo, Japan), and fixed in 10% buffered formalin. Immunohistochemical examination revealed that the LCCs were an aggregate of LAM cells that had tested positive for α-SMA, melanoma-associated antigen gp100 (immunostained by monoclonal antibody clone HMB45),  ER, PgR, and VEGF-D and were enveloped by a monolayer of VEGFR-3-positive lymphatic endothelial cells. A 3,3′-diaminobenzidine tetrahydrochloride (brown) was used as the chromogen for α-SMA, gp100, ER, and PgR, and Fast-red (red) was used for VEGF-D and VEGFR-3 (original magnification ×200). LCC: LAM cell clusters, α-SMA: Alpha-smooth muscle actin, ER: Estrogen receptor, PgR: Progesterone receptor, VEGF-D: Vascular endothelial growth factor D, VEGFR-3: Vascular endothelial growth factor receptor 3

The patient was referred to our hospital in August 2016. She had no history of smoking. Her medical history included an ectopic pregnancy and right oophorectomy (at the age of 28 years), myomectomy, and endometriosis (at the age of 34 years). Her allergic predisposition was remarkable; she had pollinosis, was allergic to cedar and yellowtail, and her history included two incidences of anaphylaxis caused by yellowtail. There was no family history of tuberous sclerosis complex. Her pulmonary function tests revealed vital capacity (VC) 3.21 L (93.6% pred), FEV1 2.04 L (73.2% pred), FEV1/FVC 66.0%, and diffusing capacity of the lungs for carbon monoxide (DLCO) 12.82 ml/mim/mmHg (53.6% pred). Serum vascular endothelial growth factor D (VEGF-D) concentration was 7,982 pg/ml (≥800 pg/ml). Right chylous pleural effusion was observed on thoracentesis and several LAM cell clusters (LCCs) were identified (Figure 2) [9], thereby leading to the diagnosis of LAM per the clinical guidelines [10].

**Figure 3:**
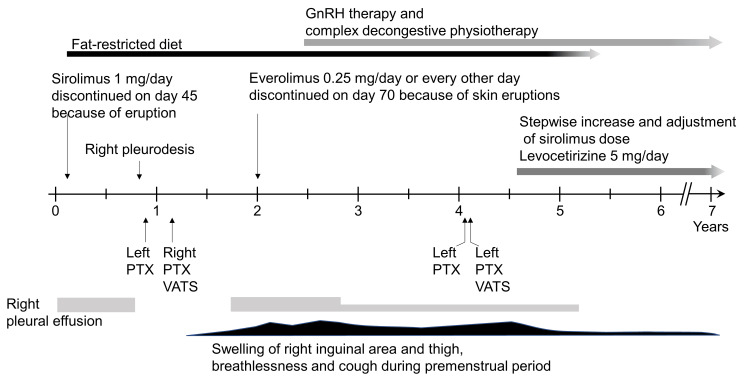
Schematic presentation of clinical course including LAM-related events, treatment, and patient condition LAM: Lymphangioleiomyomatosis, GnRH: Gonadotropin-releasing hormone analogue, PTX: Pneumothorax, VATS: Video-assisted thoracoscopic surgery

The subsequent clinical course after diagnosis has been schematically depicted in Figure 3.

**Figure 4:**
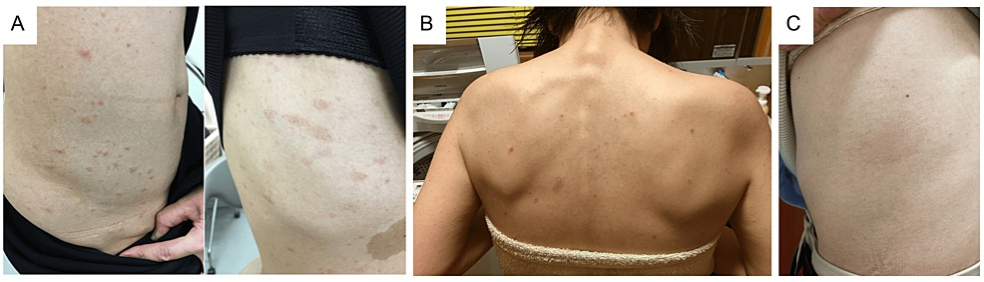
Adverse cutaneous hypersensitivity reactions to sirolimus and everolimus A: Slightly itchy erythema, some with attached collar-like scales, appeared on the trunk on day 41 when the patient was on sirolimus 1 mg/day. Sirolimus was discontinued on day 45. B: Skin rash with itchy sensation on patient’s trunk while taking everolimus 0.25 mg/day every other day and histamine H1-receptor antagonist (bepotastine besilate 20 mg/day). C: Fixed drug eruption was observed from the left lateral chest to the waist while taking sirolimus 0.6 mg/day.

Sirolimus was initiated at the dose of 1 mg/day along with a fat-restricted diet. On the 41st day following the initiation of sirolimus, round to oval erythema measuring 0.5 cm to 2 cm with attached collar-like scales, some with targetoid erythema appeared on the trunk (Figure 4 A), because of which sirolimus was discontinued on 45th day. No evidence of eosinophilia was found in the blood, and a lymphocyte stimulation test for sirolimus yielded negative results. Thereafter, the patient was followed without sirolimus.

**Figure 5:**
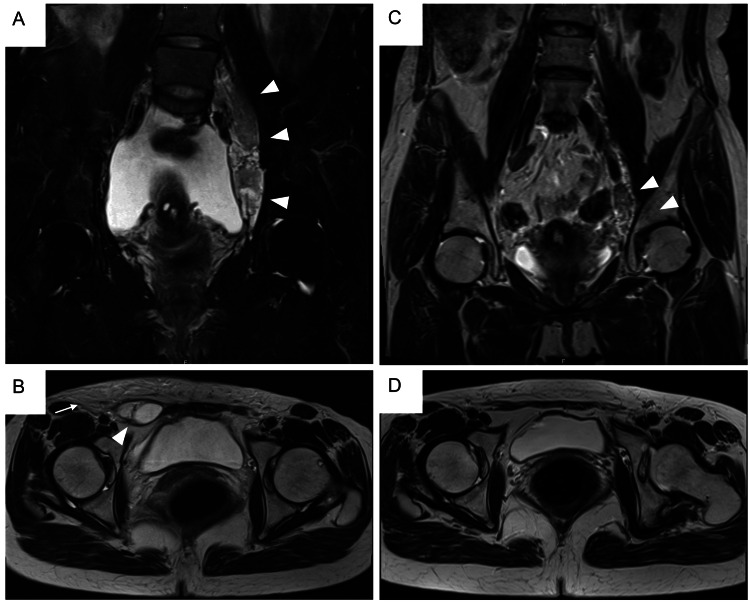
MRI images of the pelvic cavity The T2-weighted coronal (A and C) and axial (B and D) images are shown. A and B (February 2018): Pelvic retroperitoneal lymphangioleiomyomas (arrowheads in A) and lymphangioleiomyomas in the right groin area (arrowhead in B). Note lymphedema over the peri-groin subcutaneous tissue (arrow in B). C and D (August 2022): When the patient was on a dose of 0.5 mg/day of sirolimus, MRI images revealed remarkably shrunk pelvic retroperitoneal lymphangioleiomyomas (arrowheads in C) and almost complete resolution of lymphangioleiomyomas in the right groin area and lymphedema over the peri-groin subcutaneous tissue (D).

In May 2017, she had to undergo pleurodesis using 10 KE of OK-432 for a right pleural effusion (KE: Klinische Einheit to express the dose of OK-432; 1 KE of OK-432 contains 0.1 mg of dried preparation of *Streptococcus pyogenes*, group A3). In June, she suffered from a left pneumothorax. Then, in September, she had a right pneumothorax; video-assisted thoracoscopic surgery (VATS) was used for the treatment. She experienced swelling in the right groin area and thigh, which gradually deteriorated in the perimenstrual period. The MRI images obtained in February 2018, revealed ascites, the growth of retroperitoneal lymphangioleiomyomas, newly developed lymphangioleiomyomas in the right groin area, and lymphedema over the peri-groin subcutaneous tissue (Figures 5 A-B), indicating the progression of LAM.

Therefore, in July 2018, a different oral mTOR inhibitor everolimus was initiated at a negligible dose of 0.25 mg/day. Four days later the patient developed an itchy sensation, leading to the reduction in the dose of everolimus to 0.25 mg/day every alternate day and the addition of histamine H1-receptor antagonist (bepotastine besilate 20 mg/day) to the treatment regimen. However, the development of an itchy sensation with a skin rash on her trunk indicated nontolerance of even this low dose of everolimus, leading to its discontinuation on day 70 (Figure 4 B).

**Figure 6:**
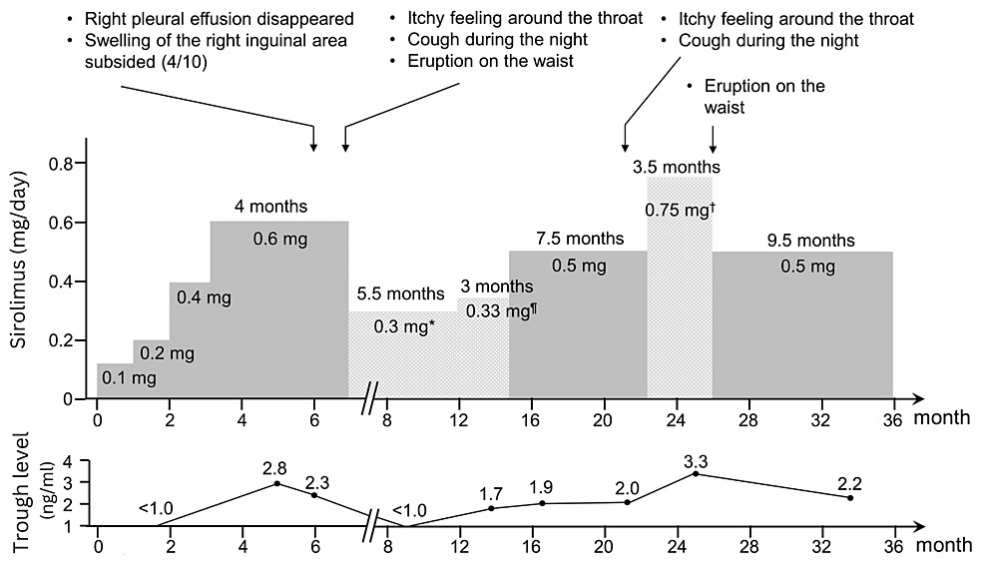
Schematic presentation of dose modification of sirolimus and trough level in blood with regards to patient’s condition The shaded area represents a dose of sirolimus as mg/day, whereas the stitched area indicates a mean dose of sirolimus as an average mg/day. *Taking 0.6 mg of sirolimus every other day; ^¶^A cycle of taking 0.5 mg/day for two days followed by one day off; ^†^Alternatively taking 1 mg/day and 0.5 mg/day

Because of mTOR inhibitor-induced allergic cutaneous reaction, a chemical oophorectomy with GnRH, i.e., subcutaneous injection of 1.88 mg leuprorelin acetate every four weeks along with complex decongestive physiotherapy was initiated in December 2018. After her menstruation had been successfully arrested in three months, her serum 17b-estradiol decreased to menopausal levels. Although her symptoms of dyspnea and swelling of the lower abdomen extending to the right groin and thighs that had exacerbated in the perimenstrual period gradually reduced, she had a recurrence of left pneumothorax in July 2020 and August 2020, which required treatment with VATS. Furthermore, she would experience an exacerbation of the swelling around the right groin and thigh as she approached the due date for her scheduled subcutaneous injection of leuprorelin. This indicated that GnRH therapy alone was not sufficient to control her condition of LAM and improve her quality of life. Based on the notion that the slowly progressive course of LAM could not be regulated without mTOR inhibitors, a decision of desensitization to sirolimus was taken with her consent after informing her of the benefits and risk of allergic reactions in January 2021 (Figure 6).

After smashing a sirolimus tablet to prepare powdered medicine, treatment was initiated at the dose of 0.1 mg/day for one month, followed by a slow increase in the dose, i.e., 0.2 mg/day for one month, 0.4 mg/day for one month, and 0.6 mg/day for four months. Moreover, a histamine H1-receptor antagonist (levocetirizine hydrochloride 5 mg/day) was included in the treatment regimen with sirolimus. The trough levels of sirolimus in blood were 2.8 ng/ml and 2.3 ng/ml after two and three months of treatment with 0.6 mg/day sirolimus, respectively. The right pleural effusion almost disappeared, no skin rash was noted, and the swelling in the right groin area had reduced to approximately 40% of the initial level (patient’s subjective scale). Because of her symptoms such as persistent itchy sensation around the throat, nocturnal cough, and an egg-sized erythema with some scaling on the lumbar region (Figure 4 C), which suggested allergic reactions to sirolimus, her dosage was reduced to 0.6 mg every other day (dosage of 0.3 mg/day in average, see Figure 6) for the subsequent six months approximately.

As the patient appeared to safely tolerate 0.5 mg/day of sirolimus, she was advised to use a pill cutter to cut pills of 1 mg sirolimus into half and continue treatment. Then, the dose of sirolimus was slowly increased; a cycle of taking 0.5 mg/day for two days followed by one day off (the dosage of 0.33 mg/day on average) for three months, followed by 0.5 mg/day for 7.5 months (see Figure 6) was initiated. An MRI performed while she was on 0.5 mg/day of sirolimus demonstrated a marked reduction in the size of the retroperitoneal lymphangioleiomyomas and the disappearance of the ascites, right inguinal lymphangioleiomyomas, and lymphedema in the subcutaneous tissue (Figures 5 C-D). Sirolimus trough levels in blood measured while on 0.5 mg/day of sirolimus were 1.9 and 2.0 ng/ml. Further attempts to increase the daily dosage of sirolimus, alternatively taking 1 mg/day and 0.5 mg/day for 3.5 months (dosage of 0.75 mg/day on average, see Figure 6), did not yield any further improvement in subjective symptoms (the trough sirolimus level was 3.3 ng/ml), and a pruritic rash appeared around her waist; therefore, the dose was reduced again to 0.5 mg/day.

The patient continues to consume a daily dose of 0.5 mg of sirolimus to this day (approximately 9.5 months). There have been no episodes of skin rash; however, there have been occasional incidences of nocturnal cough and itchy throat, although mild. The pulmonary function tests performed in June 2023 showed VC 2.98 L (89.4% pred), FEV1 2.07 L (78.1% pred), and FEV1/FVC 68.1%, DLCO 15.38 ml/min/mmHg (76.7% pred). The trough sirolimus level was 2.2 ng/ml while on 0.5 mg/day of sirolimus.

## Discussion

To date, only two case reports have previously reported rapid oral desensitization because of adverse drug reactions to mTOR inhibitors. The first case was a 27-year-old man with renal cell carcinoma, who experienced anaphylaxis after receiving temsirolimus and had to undergo rapid oral desensitization to everolimus, after which he was able to safely continue treatment with 10 mg/day of everolimus [7]. The second case involved a 41-year-old woman with LAM who developed angioedema and urticaria on her face after eight weeks of sirolimus treatment. She was treated with rapid oral desensitization to sirolimus using the same dosing schedule as that used in the first report [8]. In the case of our patient, who had a remarkable allergic predisposition, we suspected that the skin rash was caused by an allergic reaction because the same adverse cutaneous drug reactions occurred with sirolimus and everolimus. Skin rash was pruritic and occurred even at very small doses of everolimus.

We attempted to desensitize the patient to sirolimus using a stepwise approach. We slowly increased the dose schedule for the following reasons. First, for adverse drug reactions caused by antituberculosis drugs; desensitization is commonly performed by initiating with a small dose, which is followed by a gradual increase in the dose. Second, although the target trough level in the blood for sirolimus and everolimus is typically supposed to be 5 ng/m1-15 ng/m1 [2,6,11], we have previously reported that low trough levels of sirolimus, such as 1 ng/m1-2 ng/ml, are also therapeutic [12]. Moreover, it is a well-known fact that higher trough levels of mTOR inhibitors not only yield greater clinical benefits but also result in increasingly severe adverse events. Pharmacokinetic data reveals that sirolimus has a long half-life of 62 ± 16 hours (mean ± SD) in blood [13]; hence, even low levels of sirolimus in blood, which last for a long time, could not only make it beneficial in terms of gradual desensitization but is also associated with a reduced risk of allergic reactions. Third, the results of the Exist-3 study which involved the comparison of high-dose and low-dose groups of everolimus on seizures, demonstrated that the high-dose group exhibited a faster onset of effect, but the low-dose group had almost similar efficacy in controlling seizures if the drug was taken for a long period [14]. Although the low-dose group required a longer period to attain effects similar to the high-dose group, the frequency and severity of adverse events were lesser and patients demonstrated improved tolerability. Fourth, the Multicenter Lymphangioleiomyomatosis Sirolimus Trial for Safety study demonstrated a gradual decrease in the number and severity of sirolimus-induced adverse events in the two-year trial period, suggesting that the body undergoes an “adaptation for mTOR inhibitors” while on sirolimus [6]. Fifth, the quality of life of the current patient was impaired mainly by chylous pleural effusion and ascites and retroperitoneal and inguinal lymphangioleiomyomas. Furthermore, even though desensitization to sirolimus through identifying the appropriate dose of sirolimus that would balance therapeutic effects and adverse events was time-consuming, the decline of lung function expected to occur due to the progression of LAM was not expected to be severe but was within acceptable limits.

Adverse cutaneous reactions are most frequently associated with many molecular-targeted drugs currently used in the field of oncology, and are often dose-dependent and require dose modification or discontinuation [15]. The mTOR inhibitors are one of the molecular-targeted drugs used to treat malignant tumors such as renal cell carcinomas, neuroendocrine tumors, and breast cancers. We diagnosed that the skin rash in our patient was caused by an allergic reaction to mTOR inhibitors and desensitized her to the drug by initially exposing her to low doses of the drug. However, cutaneous adverse reactions in our patient appeared when the dose of sirolimus exceeded approximately 0.5 to 0.6 mg/day, possibly indicating this dosage to be a threshold for cutaneous toxicity. Under physiological conditions, mTOR is ubiquitously expressed in cells throughout the body and maintains cellular homeostasis by fluctuating its activity very sharply to maintain growth, proliferation, and metabolism in response to extracellular stimuli and internal cellular nutritional conditions [5]. The mTOR inhibitors function not only as an immunosuppressant but also as a master regulator of inflammation through sustained feedback between immune cells and stromal cells infiltrating tissues in chronic inflammation such as autoimmune diseases [15,16]. Therefore, we cannot rule out the possibility that the skin rash observed in our patient was not caused merely by an allergic reaction but could have been partly caused by on-target cutaneous toxicities of mTOR inhibitors on skin tissues. Successful desensitization of the hypersensitivity reaction of the patient can be indicated by the fact that she not only initially tolerated small doses of sirolimus but also tolerated gradually increasing doses of the drug. Furthermore, her tolerance to the drug at the initial and later dosages could indicate that the patient’s endogenous allergic predisposition was 'adapted'. Notably, we were able to figure out the appropriate dose of sirolimus that did not manifest any adverse cutaneous reaction and was therapeutic for the treatment of LAM.

## Conclusions

Herein, we reported our experience of a patient with LAM who was initially allergic to mTOR inhibitors, sirolimus, and everolimus, but later successfully tolerated a low dose of sirolimus that was still therapeutic. Although successful rapid oral desensitization to mTOR inhibitors has recently been reported, this case study demonstrated that desensitization to sirolimus could be achieved by a 'slow dose-increasing schedule', which starts with a very small dose and is followed by a careful stepwise increase. As mTOR inhibitors are the only approved drugs with scientific evidence for treating LAM and work therapeutically even at lower trough levels than the currently prevailing target range of 5 ng/ml to 15 ng/ml, it is imperative to make the effort to find the dose that balances adverse events and therapeutic effects for the benefit of these patients.

## References

[1] Goncharova EA, Goncharov DA, Eszterhas A (2002). Tuberin regulates p70 S6 kinase activation and ribosomal protein S6 phosphorylation. A role for the TSC2 tumor suppressor gene in pulmonary lymphangioleiomyomatosis (LAM). J Biol Chem.

[2] McCormack FX, Inoue Y, Moss J (2011). Efficacy and safety of sirolimus in lymphangioleiomyomatosis. N Engl J Med.

[3] Harari S, Cassandro R, Chiodini I, Taveira-DaSilva AM, Moss J (2008). Effect of a gonadotrophin-releasing hormone analogue on lung function in lymphangioleiomyomatosis. Chest.

[4] Gupta N, Lee HS, Ryu JH (2019). The NHLBI Lam registry: prognostic physiologic and radiologic biomarkers emerge from a 15-year prospective longitudinal analysis. Chest.

[5] Liu GY, Sabatini DM (2020). mTOR at the nexus of nutrition, growth, ageing and disease. Nat Rev Mol Cell Biol.

[6] Takada T, Mikami A, Kitamura N (2016). Efficacy and safety of long-term sirolimus therapy for Asian patients with lymphangioleiomyomatosis. Ann Am Thorac Soc.

[7] Karlin E, Allinder JM, Dworski R, Stollings JL (2014). Rapid oral desensitization to everolimus. Ann Pharmacother.

[8] Sebaaly J, Bowers L, Mazur J, Kotloff R, Olmsted BR, Kaplan A, Strange C (2016). Rapid oral desensitization to sirolimus in a patient with lymphangioleiomyomatosis. J Allergy Clin Immunol Pract.

[9] Mitani K, Kumasaka T, Takemura H (2009). Cytologic, immunocytochemical and ultrastructural characterization of lymphangioleiomyomatosis cell clusters in chylous effusions of patients with lymphangioleiomyomatosis. Acta Cytol.

[10] Gupta N, Finlay GA, Kotloff RM (2017). Lymphangioleiomyomatosis diagnosis and management: high-resolution chest computed tomography, transbronchial lung biopsy, and pleural disease management. An official American Thoracic Society/Japanese Respiratory Society clinical practice guideline. Am J Respir Crit Care Med.

[11] French JA, Lawson JA, Yapici Z (2016). Adjunctive everolimus therapy for treatment-resistant focal-onset seizures associated with tuberous sclerosis (EXIST- 3): a phase 3, randomised, double-blind, placebo-controlled study. Lancet.

[12] Ando K, Kurihara M, Kataoka H (2013). Efficacy and safety of low-dose sirolimus for treatment of lymphangioleiomyomatosis. Respir Investig.

[13] Klawitter J, Nashan B, Christians U (2015). Everolimus and sirolimus in transplantation-related but different. Expert Opin Drug Saf.

[14] Franz DN, Lawson JA, Yapici Z (2021). Adjunctive everolimus therapy for tuberous sclerosis complex-associated refractory seizures: Results from the postextension phase of EXIST-3. Epilepsia.

[15] Macdonald JB, Macdonald B, Golitz LE, LoRusso P, Sekulic A (2015). Cutaneous adverse effects of targeted therapies: part II: inhibitors of intracellular molecular signaling pathways. J Am Acad Dermatol.

[16] Suto T, Karonitsch T (2020). The immunobiology of mTOR in autoimmunity. J Autoimmun.

